# Design of SnO_2_ Aggregate/Nanosheet Composite Structures Based on Function-Matching Strategy for Enhanced Dye-Sensitized Solar Cell Performance

**DOI:** 10.3390/ma11091774

**Published:** 2018-09-19

**Authors:** Dongting Wang, Shangheng Liu, Mingfa Shao, Jinghan Zhao, Yukun Gu, Qiuyi Li, Xianxi Zhang, Jinsheng Zhao, Yuzhen Fang

**Affiliations:** Shandong Provincial Key Laboratory of Chemical Energy Storage and Novel Cell Technology, School of Chemistry and Chemical Engineering, Liaocheng University, Liaocheng 252059, China; liush@lcu.edu.cn (S.L.); shaomf@lcu.edu.cn (M.S.); zhaojh@lcu.edu.cn (J.Z.); guyk@lcu.edu.cn (Y.G.); liqy@lcu.edu.cn (Q.L.); zhangxianxi@lcu.edu.cn (X.Z.); zhaojinsheng@lcu.edu.cn (J.Z.); fangyuzhen@lcu.edu.cn (Y.F.)

**Keywords:** hierarchical SnO_2_ aggregates, room temperature method, SnO_2_ aggregate/nanosheet composite, photoanode, dye-sensitized solar cells

## Abstract

Hierarchical SnO_2_ nanocrystallites aggregates (NAs) were prepared with a simple room temperature–based aqueous solution method followed by simple freeze-drying treatment. The as-prepared SnO_2_ NAs were subsequently combined with SnO_2_ nanosheet–based structures from the viewpoint of a function-matching strategy, and under an optimized condition, a power conversion efficiency (PCE) of 5.59% was obtained for the resultant hybrid photoanode, a remarkable 60% enhancement compared to that of dye-sensitized solar cells (DSCs) fabricated with bare SnO_2_ NAs architecture. The significantly enhanced efficiency can be attributed to the combination of the desirable electron transport property obtained by the intentionally introduced SnO_2_ nanosheets (NSs) and the effectively retained inherent characteristics of SnO_2_ NAs, i.e., large surface area and strong light-scattering effect. This work provides a promising approach for the rapid development of highly efficient SnO_2_ photoanode film-based DSCs with the properties of simplicity of operation and control over the photoanode composition.

## 1. Introduction

Since the breakthrough work in mesoscopic dye-sensitized solar cells (DSCs) by O’Regan and Grätzel, a great deal of effort has been made on this unique device because of its advantages of being highly efficient and environmentally friendly, with low cost and ease of fabrication [1,2,3]. Apart from the most commonly studied photoanode material, TiO_2_, alternative metal oxides such as SnO_2_ have been widely investigated in the past decade. In contrast to TiO_2_, SnO_2_ has two outstanding advantages, a larger band gap (Eg = 3.6 eV) and faster mobility of charge carriers (~100–200 cm^2^V^−1^s^−1^) within individual structures [4,5,6], which results in the long-term stability of DSCs and reduced charge recombination probability within the photoanode film. For SnO_2_-based cells, the conventional photoanode film is generally composed of mesoporous nanoparticles, hence it provides a large surface area for sufficient dye loading. However, a major drawback to be overcome for such SnO_2_-based DSCs is the severe charge carrier recombination losses in the electron transfer process as a result of various interfaces and grain boundaries [7,8,9]. In addition, the weak visible light-scattering capability of such porous photoanode has been proven to be another disadvantage, since a large portion of the incident light will directly pass through the small nanoparticle-based film without effective harvesting and utilization [10,11]. As such, although SnO_2_ nanoparticle-based DSCs have been extensively investigated, the conversion efficiencies are still much lower than those of TiO_2_, making them less competitive. Therefore, how to effectively accelerate the electron transfer and enhance the visible light-scattering ability of SnO_2_ photoanode film with retention of relatively high surface area is regarded as the key approach to attain DSCs with high conversion efficiency.

Integrating the small-sized nanoparticles into three-dimensional (3D) microstructures such as aggregates and hollow spheres has proven to be a promising strategy for efficiency enhancement, since the 3D structures not only offer large surface areas for sufficient dye molecule loading favored by the small size of the constructed building blocks, but also improve the light-scattering capability provided by these architectures, with tunable size ranging from hundreds of nanometers to micrometers [12,13,14]. To date, the DSCs assembled with specific 3D-nanostructure photoanode film has achieved the highest conversion efficiency of 8.21% after TiCl_4_ treatment [15]. Nevertheless, small inner grains in the 3D structures and poor interconnectivity between these microarchitectures hinders electron transport within the photoanode film and thus inflicts charge recombination to some extent [16,17]. Compared with the 3D architectures, two-dimensional (2D) nanosheets (NSs) could provide straight electric pathways for faster charge transport due to their excellent electrical conductivity and increased electron diffusion length, thus preventing the recombination losses originating from the reaction between oxidizing dyes and photogenerated electrons [18,19]. However, the weak light-harvesting efficiency of these films, ascribed to the small specific surface area and moderate light-scattering capability, constrains their power conversion efficiency to relatively low levels [20,21]. Moreover, the 2D nanostructures can significantly agglomerate or be overlaid during the film preparation process, leading to reduced surface area and/or light-scattering capability. Actually, it has been established that the application of single component or morphology for photoanode film makes it hard to simultaneously satisfy the required properties—high specific surface area, outstanding light scattering, and fast electron transport—for efficient DSCs due to the intrinsic drawbacks of each element. With regard to these issues, substantial efforts have been focused on fabricating multidimensional hybrid photoanode nanomaterials, aiming to combine the desirable characteristics of each nanostructure. As an example, Yang et al. successfully formed SnO_2_/TiO_2_ heterostructure by incorporating 2D SnO_2_ nanosheets into TiO_2_ nanoparticle-based photoanodes and found that in this system, rapid electron transport pathways and improved light scattering were obtained from 2D SnO_2_ nanosheets with the retention of sufficient dye-loading ability [22]. Moreover, Tao et al. reported a novel ZnO composite photoanode composed of 3D ZnO aggregates (NAs) and bridging 2D ZnO nanosheets (NSs) that yielded a near-record power conversion efficiency (PCE) of 7.35% because of the effective combination of the relatively strong light-scattering property of ZnO NAs with the increased surface area and favorable electron transport of ZnO NSs [23]. In line with these noteworthy features of the composite photoanode, SnO_2_ hybrid photoanode consisting of function-matching structures shows great promise in dramatically enhancing DSC efficiency. However, to date, the design and investigation of SnO_2_ hybrid photoanode has yet to be reported.

Herein, for the first time, SnO_2_ NS–based 3D and 2D sheet composite architectures are intentionally introduced in 3D SnO_2_ NAs film to construct hybrid SnO_2_ architecture, within which 3D SnO_2_ NAs act as building blocks to augment the accessible surface area for sufficient dye uptake and enhance light scattering for effective light utilization, while SnO_2_ NSs mainly play the vital role of facilitating electron transport within the film. To coordinate the specific features of each component, the ratio of NAs to NSs is rationally tuned, and under optimum conditions, an enhanced photovoltaic conversion efficiency of 5.59% was obtained for the composite photoanode, showing a remarkable 60% increment in comparison with the DSC fabricated with bare SnO_2_ NAs under the same conditions. Various measurements revealed that such outstanding performance of the resultant photoanode was ascribed to a reasonable combination of the excellent charge transport and strong light-harvesting capabilities resulting from the synergy of SnO_2_ NAs architecture and SnO_2_ nanosheet-based structures.

## 2. Experimental Section

### 2.1. Materials

Tert-butylpyridine (t-BPy), 3-methoxypropionitrile, iodide (I_2_), anhydrous lithium iodide (LiI), and 2,3-dimethyl-1-propyl imidazolium iodide (DMPII) were purchased from (Sigma, St. Louis, MO, USA). Na_2_CO_3_, SnF_2_, SnCl_2_·2H_2_O, C_6_H_12_N_4_ (methenamine), TiCl_4_, and H_2_PtCl_6_·6H_2_O were all obtained from commercial sources and used without further treatment. The popular dye sensitizer [cis-bis(isothiocyanato)bis-(2,2-bipyridyl-4,4-dicarboxylato) ruthenium (II) bis(tetrabutyl-ammonium)] was purchased from (Dyesol, New South Wales, Australia).

### 2.2. Preparation of SnO_2_ Aggregates

SnO_2_ aggregates were prepared by hydrolyzing SnCl_2_ at room temperature, followed by freeze-drying. In a typical synthesis, 1.128 g SnCl_2_·2H_2_O was first dissolved in deionized water (40 mL) under magnetic stirring, followed by the slow addition of 1.25 mM Na_2_CO_3_. Still at room temperature, the resultant white suspension was continuously stirred for 7 days, leading to the formation of homogeneous SnO_2_ solution. Afterwards, the resulting solution was subjected to freeze-drying for 24 h, followed by repeated centrifugation/sonication/dispersion in water 5 times. After drying at 80 °C for 4 h in air, SnO_2_ aggregates were finally obtained.

### 2.3. Preparation of SnO_2_ Nanosheets

SnO_2_ NSs were prepared with a hydrothermal method similar to the literature reported by other groups [19]. Typically, 0.047 g SnF_2_ was first dissolved in deionized water (30 mL), followed by the addition of 0.035 g methenamine under magnetic stirring. After stirring for 30 min, the obtained white turbid suspension was transferred into a Teflon-lined stainless steel autoclave (60 mL) and heated at 180 °C for 12 h in an electric oven. After being cooled to room temperature, the obtained brown suspension was repeatedly washed with deionized water and ethanol by centrifugation at a rate of 9000 rpm for 10 min. Finally, the product was harvested and dried at 60 °C overnight under ambient conditions.

### 2.4. Preparation of SnO_2_ Hybrid Photoanode and Cell Assembly

The SnO_2_ hybrid electrode was formed by coating SnO_2_ composite on precleaned fluorine-doped tin oxide (FTO)-coated glass plates (Nippon, Tokyo, Japan, 7 Ω/sq) in conjunction with a programmed annealing procedure [24]. As an example, 50 wt% (the content of NS in the mixture) SnO_2_ hybrid photoanode film was obtained as follows: NAs and NSs with a total weight of 0.2 g were dispersed into a mixture of ethyl cellulose (0.1 g), terpineol (0.6 g), ethanol (2 mL), and acetic acid (0.04 mL), followed by grounding for approximately 60 min. The obtained viscous paste was spread uniformly on FTO substrate with the typical doctor-blading technique. The thickness of the films was controlled by the layers of tape adhered on the FTO substrate. For all samples, the same number of layers of adhesive tape was applied to ensure identical thickness of working electrodes. After being dried, the photoanode films were formed by gradually annealing in a programmed procedure, first at 325 °C for 5 min, then at 375 °C for 5 min, then at 450 °C for 15 min, and finally at 500 °C for 15 min. After that, the synthesized SnO_2_ film was treated with 0.4 M TiCl_4_ aqueous solution at 77 °C for 50 min, washed with water and ethanol to remove unanchored Ti source, and finally sintered at 450 °C for 2 h. In parallel, another 4 samples with different NS contents (0 wt%, 33 wt%, 67 wt%, and 100 wt%) were prepared under the identical synthesis procedures as described above. For simplicity, the above 5 as-prepared samples are denoted as TP, TPS1, TPS2, TPS3, and TPS4, respectively.

Dye sensitization was performed by soaking the resultant SnO_2_ hybrid electrodes in 5.0 × 10^−4^ M N719 dye ethanolic solution for 24 h in the dark at room temperature. The Pt counter electrode was obtained by dropping 0.35 mM H_2_PtCl_6_/isopropanol solution on the FTO conductive substrate and subsequently annealing in a muffle furnace at 400 °C for 30 min under air flow conditions. The DSCs were then assembled by bonding the sensitized photoanode (0.16 cm^2^) and prepared Pt-coated counter electrode together with a hot-melt Surlyn film (25 μm thick; DuPont, Wilmington, DE, USA). The liquid electrolyte, consisting of 0.1 M LiI, 1.0 M DMPII, 0.12 M I_2_, and 0.5 M t-BPy in 3-methoxypropionitrile, was filled into the whole internal space of the fabricated cells by vacuum backfilling.

### 2.5. Characterizations

XRD patterns were recorded from a D/MAX-rA diffractmeter (Rigaku, Tokyo, Japan) with a Cu Kα radiation source (λ = 0.15406 nm). The morphologies of the as-prepared SnO_2_ NAs and NSs and the resultant hybrid films were examined with transmission electron microscopy (TEM, JEOL-2010, Hitachi, Tokyo, Japan) and field-emission scanning electron microscope (FESEM, JEOL-6701F, Akishima, Japan), respectively. The specific surface areas (S_BET_) were analyzed based on nitrogen adsorption–desorption isotherms recorded with an Autosorb iQ-XR Analyzer (Quantachrome Instruments, Boynton Beach, FL, USA). Dye desorption experiments were conducted by immersing the photoanode films in 1.0 M NaOH in water/ethanol (50:50, *V*/*V*) solution, and the concentration of desorbed dye was quantitatively analyzed using an ultraviolet-visible (UV–vis) spectrophotometer (Cary 500, Varian, Palo Alto, CA, USA). The diffuse reflectance spectra of the films with various NS content were investigated with a QEX10 spectral response system from PV Measurements, Inc. The photoelectrochemical characterizations were performed by a Keithley Model 2400 source meter under simulated AM 1.5 G one-sun illumination (100 mW·cm^−2^, Newport Corporation, Los Angeles, CA, USA) with the sweep direction from forward to reverse. Before each test, the light intensity was first calibrated by a National Renewable Energy Laboratory (NREL) calibrated Si solar cell (PV Measurements, Inc., Boulder, CO, USA). Electrochemical impedance spectroscopy (EIS) experiments were performed under one-sun illumination using an electrochemical workstation (CHI760, CH Instruments, Shanghai, China) at V_OC_ with an AC modulation signal of 10 mV within a frequency range of 0.1–10^5^ Hz.

## 3. Results and Discussion

### 3.1. XRD Patterns and BET Surface Area Analysis

**Figure 1:**
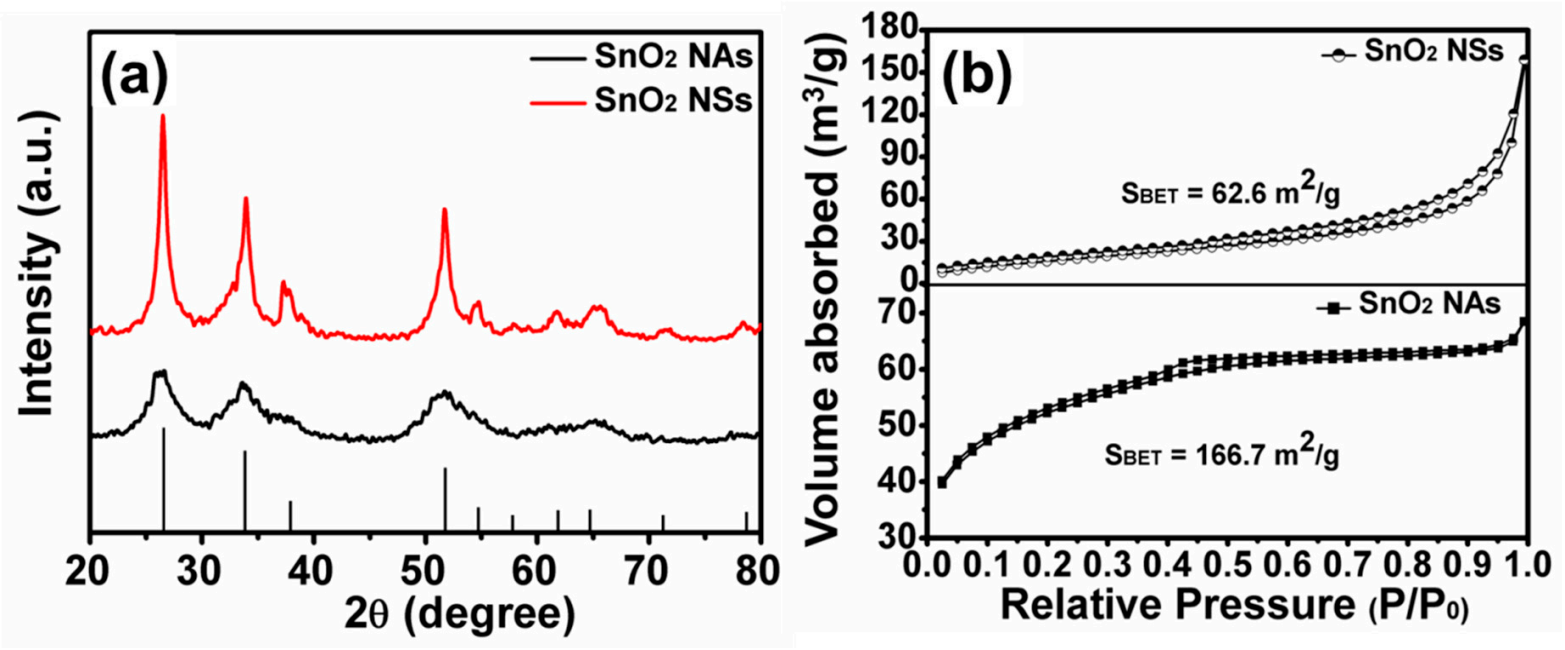
(**a**) XRD patterns of the as-prepared SnO_2_ NAs and SnO_2_ NSs. (**b**) Nitrogen adsorption and desorption isotherms of SnO_2_ NAs and SnO_2_ NSs. Vertical bars below represent the standard XRD pattern of SnO_2_.

The crystalline phases of both SnO_2_ NAs and NSs were analyzed with X-ray diffraction (XRD) spectra. As shown in Figure 1a, it can clearly be seen that all the diffraction peaks of the products are well assigned to the tetragonal rutile structure of SnO_2_ (JCPDS. 41–1445), and no other impurity is detected, suggesting the successful transformation from Sn^2+^ to SnO_2_ during the room temperature stirring process. As reported by Xie et al., Sn^2+^ can be oxidized to Sn^4+^ in aqueous solution in the presence of oxygen, and the first formed amorphous phase can crystallize further at room temperature due to its inherently strong tendency to transform to crystalline structure [25,26]. Moreover, compared with the common SnO_2_ samples suffering from high-temperature calcination, the synthesized product shows much broader diffraction peaks, indicating the small size of primary nanoparticles or building blocks in the as-prepared SnO_2_ NA product. As calculated on the basis of Scherer’s formula, the average crystallite size of the primary nanoparticles determined by the full width at half-maximum (FWHM) of the (110) peak is about 5 nm. The above morphological feature of SnO_2_ NA finally enables the desired specific surface area. As shown in Figure 1b, N_2_ adsorption and desorption isotherms evidence that SnO_2_ NAs present an impressively high specific surface area of approximately 166.7 m^2^/g, which is twice that of SnO_2_ NSs (62.6 m^2^/g), suggesting the potentially sufficient dye-anchoring ability of the resultant photoanodes.

### 3.2. Morphological and Composition Characterization

**Figure 2:**
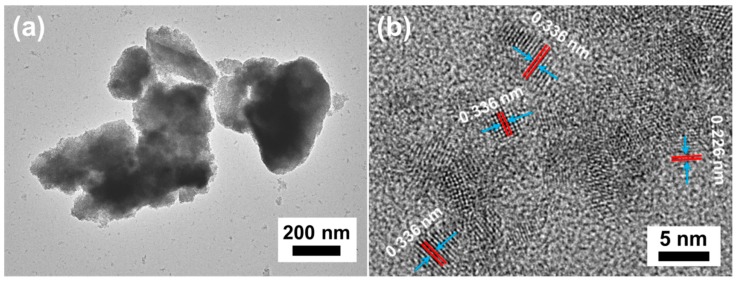

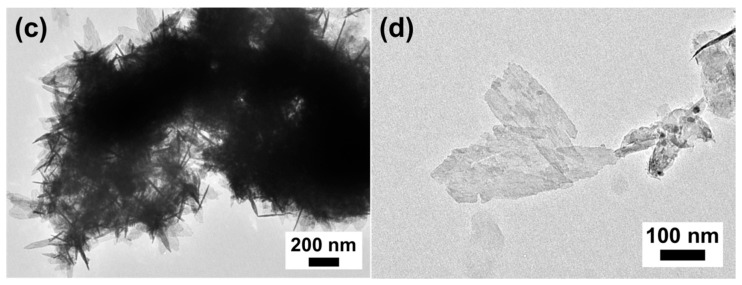
(**a**) TEM and (**b**) HRTEM images of synthesized SnO_2_ NAs, and (**c**,**d**) TEM images of 3D structured SnO_2_ NSs and random SnO_2_ NSs.

As can be seen from Supplementary Figure S1, the as-synthesized SnO_2_ NAs show irregularly aggregated character with size ranging from about 100 nm to 5 μm. The amplified TEM image (see Figure 2a) distinctly evidences that the SnO_2_ NAs are hierarchically composed of numerous tiny nanoparticles. The formation of specific features could result from the given ratio of SnCl_2_ and Na_2_CO_3_ as suggested in our previous work [23]. Further HRTEM characterization, shown in Figure 2b, reveals clear lattice fringes of *d* = 0.26 and 0.34 nm, respectively, corresponding well to the lattice spacing of (101) and (110) planes of rutile SnO_2_ lattice. Moreover, the crystal size demonstrated in the HRTEM image exhibits good consistency with the calculated data from the XRD characterization.

Apart from SnO_2_ NAs, another typical nanostructure, SnO_2_ NSs, was also synthesized and the morphology of the product was characterized. As demonstrated in Figure 2c, the typical TEM image proves the successful formation of tightly interconnected nanosheets based on a flower-like 3D hierarchical structure. These outstanding features should be not only favorable for the penetration of liquid electrolyte due to the formation of a highly open and porous structure, but also advantageous for light scattering due to the comparable size of SnO_2_ NSs with the wavelength of the incident light [27]. More interestingly, random 2D SnO_2_ nanosheets with high transparency are also obtained (see Figure 2d). Such morphological characteristics indicate that the formed nanosheets could effectively connect the SnO_2_ NAs framework and serve as an electron transport highway, thus shortening the diffusion pathways and lengthening the diffusion distance [22,28].

**Figure 3:**
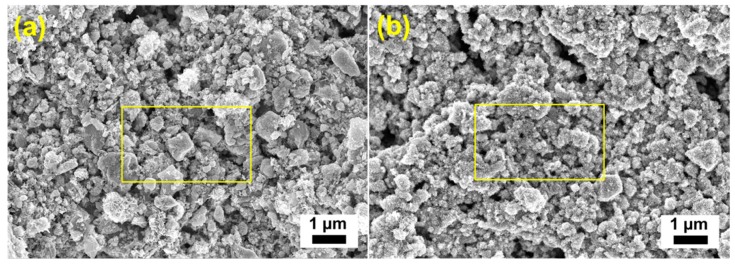

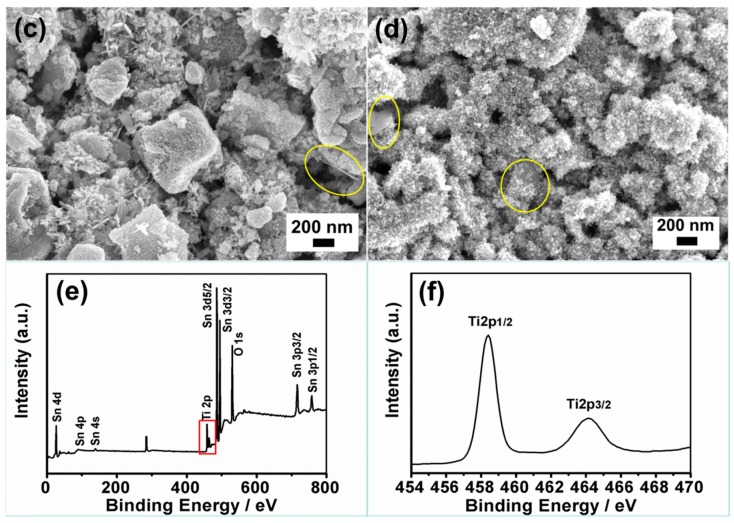
SEM images of SnO_2_ NA/NS hybrid films (**a**,**c**) before and (**b**,**d**) after TiCl_4_ treatment with different modifications; XPS spectra of (**e**) survey and (**f**) Ti 2p peaks.

After mixing the synthesized SnO_2_ NAs together with the SnO_2_ NSs, hybridized films with and without TiCl_4_ treatment were fabricated, and the corresponding SEM images are displayed in Figure 3. It can be seen from Figure 3a that both hierarchical SnO_2_ NSs and SnO_2_ NAs can be easily visualized before TiCl_4_ treatment, implying the well-preserved inherent characteristics of each composition. Further observation reveals that not only hierarchical SnO_2_ NSs but also 2D SnO_2_ NSs can be clearly seen inside the SnO_2_ hybridized film (see Figure 3c), which is consistent with the above TEM characterization. As can be seen in Figure 3b,d, after TiCl_4_ treatment, the surface of the TPS2 becomes obviously rougher and the size seems much larger in comparison with untreated film, indicating the significant influence of TiCl_4_ post treatment on the SnO_2_ film structure. However, it is worth noting that even after being subjected to TiCl_4_ treatment, the hierarchical architecture is maintained. Additionally, a discernible detached sheet without complete coverage after TiCl_4_ treatment can be clearly observed, further confirming the presence of SnO_2_ NSs in the TiO_2_-coated SnO_2_ hybrid film.

To probe the composition of the TSP2 film, the typical XPS survey spectrum was collected. As shown in Figure 3c, the appearance of peaks corresponding to Ti, O, and Sn demonstrates the successful coating of TiO_2_ on the SnO_2_ surface. In addition, the high-resolution XPS spectrum with scanning over the above Ti element was conducted and the result is depicted in Figure 3d. Notably, the Ti 2p XPS spectrum shows two typical peaks at 458.4 eV and 464.2 eV, corresponding to the binding energies of different Ti^4+^ states, i.e., Ti 2p 3/2 and Ti 2p 1/2, respectively [29,30].

### 3.3. Photocurrent Density−Voltage Characteristics

**Figure 4:**
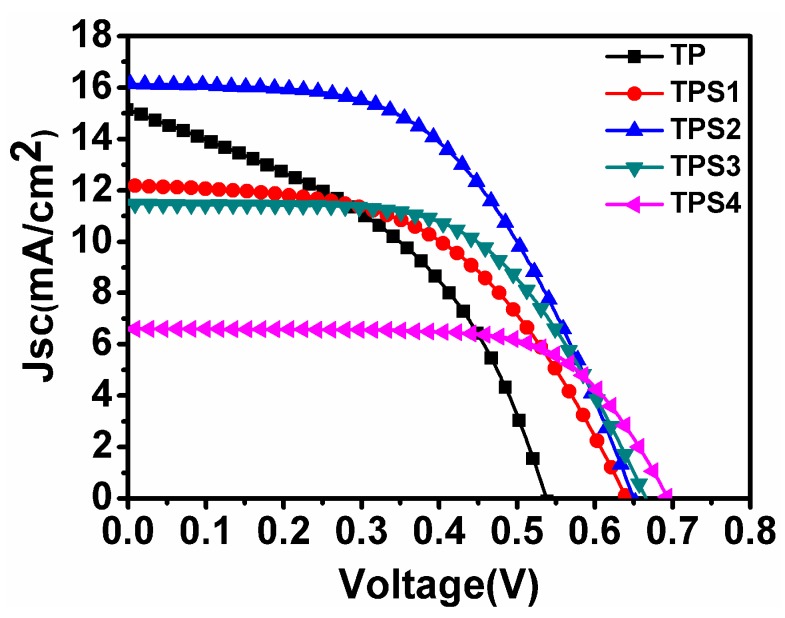
J–V characteristic curves of various DSCs based on TP, TPS1, TPS2, TPS3, and TPS4.

The photovoltaic performance of DSCs with different NS content was measured under AM 1.5 sunlight illumination (100 mW cm^−2^). The results are given in Figure 4 in the form of representative J–V curves, and detailed photovoltaic parameters (J_SC_, V_OC_, PCE, and fill factor (FF)) are shown in Table 1. As can be seen from Figure 4, the sample without NS (sample TP) exhibits a relatively high J_SC_ of 15.12 mA cm^−2^ but a low V_OC_ of 0.537 V and a FFof 0.43. This observation can be associated with the component of the working electrode. As revealed in Figure 2a, the as-prepared hierarchically structured SnO_2_ NAs most likely possess strong light-scattering effect and sufficient dye-loading capability, thus promoting photon absorption to enhance the J_SC_ value. However, the low V_OC_ and FF in turn illustrate the intrinsic disadvantage of the as-prepared SnO_2_ NAs. In contrast, the pure SnO_2_ NS–based DSC (TPS4) prepared under identical conditions shows adverse characteristics, with a high V_OC_ of 0.693 V and an impressive FF of 0.68, but a small J_SC_ of 6.59 mA cm^−2^. As reported previously, the V_OC_ could probably change with altered morphologic and/or crystallographic orientation due to the shift of the Fermi level [31,32,33]. As a typical low-index crystal facet with fewer crystal edges and defects, nanosheets could bring about increased electron density in the conduction band because of the decreased electron−hole recombination arising from the excellent electron transport property of the specific structure, thus making the negative shift of the Fermi level position. In view of the prominent advantage of nanosheets, the great improvement in photovoltaic performance is worth looking at for NSs containing hybrid structure. As expected, with the increase of NS content from 0% to 50%, the key parameters of the cells, namely TPS1 and TPS2, improve gradually, leading to a continuous increment of PCE from 3.50% to 5.59%. However, when the NS content is further increased, sample TPS3 shows drastically decreased J_SC_ and PCE (J_SC_: 11.46 mA cm^−2^; PCE: 4.48%). The above observation can be explained as follows: though the NS-based structures in the photoanode film can act as bridges to connect the interparticles, the increased NS in the hybrid film could also result in a gradual decrease of the dye-loading amount owing to their lower specific surface area as compared to SnO_2_ NAs, leading to a gradual reduction of light-harvesting capability. Consequently, when the improved electron transfer property cannot compensate the loss of surface area, low J_SC_ and PCE are expected. Therefore, it can be deduced that controlling the content of NS in the composite film is important for reasonable coordination of electron transport and light harvesting.

### 3.4. Dye Absorption and Diffuse Reflectivity

To reveal the mechanism behind the improved photovoltaic performance of the hybrid structure DSCs, the dye uptake in all photoanodes was studied. As shown in Table 1, the dye adsorption ability of the resultant SnO_2_ films is largely dependent on the NS content. Specifically, the dye-loading amount first increases from 1.94 × 10^−7^ mol cm^−2^ to 2.23 × 10^−7^ mol cm^−2^ by increasing the NS content from 0% to 33%, and then decreases from 2.07 × 10^−7^ mol cm^−2^ to 1.76 × 10^−7^ mol cm^−2^ when the NS content is increased from 50% to 67%. Another reference photoanode film, TPS4, displayed the lowest dye-loading amount, 1.15 × 10^−7^ mol cm^−2^. This significant variation in dye absorption could be explained as follows: on the one hand, the introduced SnO_2_ NSs likely disperse between SnO_2_ NAs (see Figure 3c) and inhibit their aggregation; on the other hand, the presence of SnO_2_ NAs could also retard the undesired overlap of the SnO_2_ NSs. Therefore, the hybrid structure with proper SnO_2_ NSs could offer sufficient contact area for TiO_2_ deposition or coating, which would lead to a large amount of dye absorption. However, further augmenting the content could result in severe stacking of SnO_2_ NSs, leading to reduced surface area.

**Figure 5:**
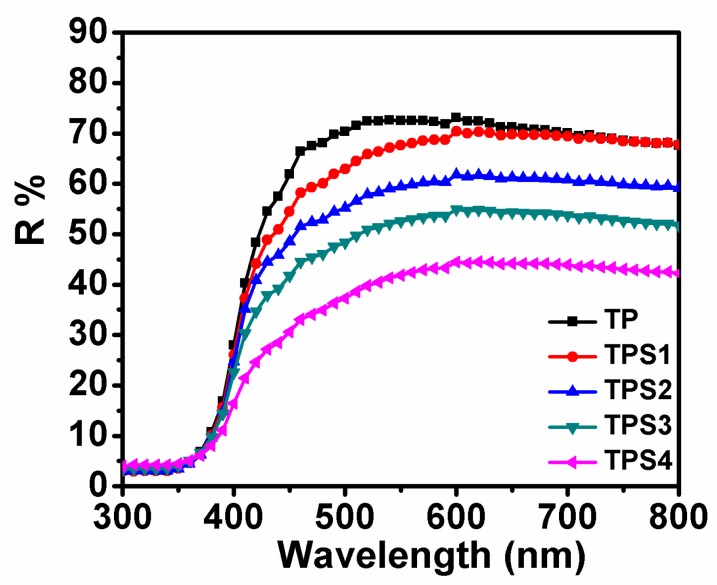
Diffuse reflectance spectra of the TP, TPS1, TPS2, TPS3, and TPS4 films.

To investigate the influence of SnO_2_ NSs on the scattering effect of the hybrid structures, the reflectivity of each photoanode film was studied, shown in Figure 5. Apparently, TP has the highest diffuse reflection, in the range of 400 nm to 800 nm, while TS shows the lowest reflectance in the same region, which actually is consistent with our hypothesis on the more effective incident light-scattering ability of SnO_2_ NAs. As for the hybrid film, increasing the amount of SnO_2_ NSs from TPS1 to TPS3 generated gradually weakened light-scattering capabilities in the entire visible light region. The obvious light reflectance intensity distinction must be ascribed to the inferior incident light-scattering capability of bare SnO_2_ NSs compared to bare SnO_2_ NAs. The above observation along with the dye desorption test confirm the outstanding features of SnO_2_ NAs for sufficient dye absorption and excellent light scattering. However, as revealed in the photovoltaic test section, sample TPS2 rather than TP achieved the highest efficiency. Therefore, considering the mismatch between the moderate light-harvesting capability and the optimum photovoltaic performance, the significant increase in PCE of the TPS2 cell must stem from the favorable electron transport and effectively suppressed electron recombination within the film.

### 3.5. EIS Analysis

**Figure 6:**
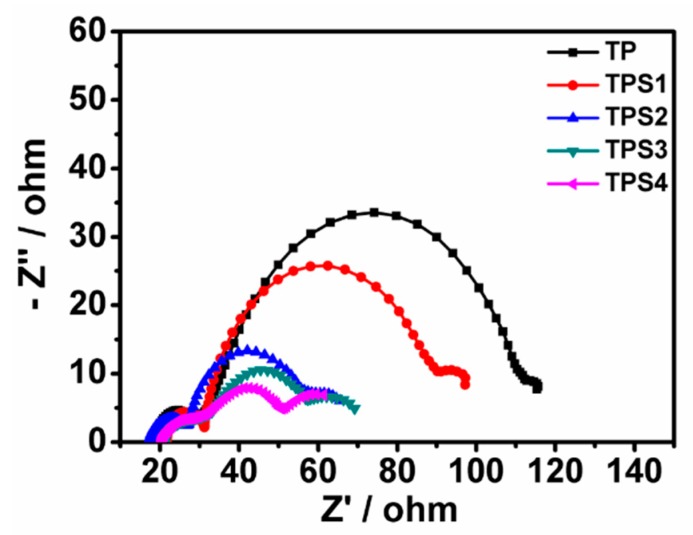
Nyquist plots of various SnO_2_ photoanode films–based DSCs.

Electrochemical impedance spectroscopy (EIS) analysis was employed to gain insight into the electrical behavior of DSCs based on SnO_2_ NAs with and without NSs, with the aim of revealing the mechanism behind the differences in photovoltaic performance among all samples. Figure 6 shows the typical EIS Nyquist plots of all DSCs obtained under a simulated solar light of 100 mW cm^−2^ at a bias potential of V_OC_. It can be seen that the EIS Nyquist plots have three well-defined semicircles in the corresponding regions, i.e., high frequency (>10^3^ Hz), medium frequency (10^0^−10^3^ Hz), and low frequency (<10^0^ Hz), which denote charge transfer resistance at the Pt counter electrode/electrolyte interface, recombination resistance at the SnO_2_/dye/electrolyte interface, and the Warburg diffusion process of I^−^/I_3_^−^ in the electrolyte, respectively [1,34]. According to the approach of Adachi et al., the charge transfer recombination impedance (R_ct_) in the photoanode can be deduced from the central semicircle of the Nyquist plot, and the corresponding resistance values can be determined by Zview software fitting [35]. As displayed in Table 1, it can be obviously seen that the charge transfer resistance of the resultant photoanodes decreases successively from 39.4 ohm for the TP to 10.2 ohm for the TPS4 cell with the increment of SnO_2_ NS amount, implying slower electron recombination for the samples with higher SnO_2_ NS content. This phenomenon is explainable, since the injected electrons could probably recombine with I_3_^−^ in the electrolyte in the SnO_2_ NA photoanode film before reaching the collector electrode (e.g., FTO substrate) due to the partial zigzag pathway for electron transfer. However, after the incorporation of SnO_2_ NS nanostructures, the produced electron could pass through the photoanode film more easily due to the effective interconnectivity between the neighboring SnO_2_ NAs and favorable electron transport route arising from SnO_2_ NSs, thus promoting electron diffusion and collection efficiency.

### 3.6. IPCE Spectra

**Figure 7:**
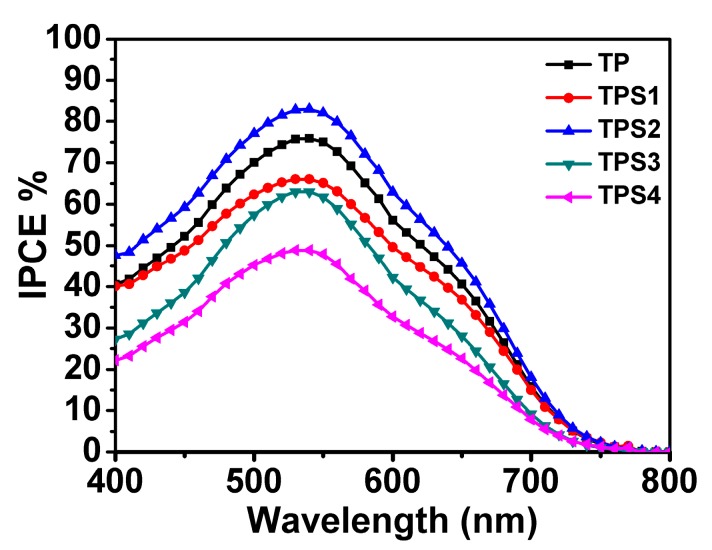
IPCE spectra of DSCs based on TP, TPS1, TPS2, TPS3, and TPS4.

Figure 7 shows the typical incident photon conversion efficiency (IPCE) spectra of various DSCs fabricated with bare SnO_2_ NAs, SnO_2_ NSs, and SnO_2_ NAs/NSs hybrid photoelectrodes as a function of the illumination wavelength ranging from 400 nm to 800 nm, in which maximum intensities for all DSCs are observed at the wavelength of 530 nm, which is consistent with the absorption characteristic of the N719 molecule. For the hybrid structures, it can be seen that the IPCE value first increases and then decreases with the increment of NS; that is, the IPCE of TPS1 and TPS2 hybrid films is much higher than that of TP-based film in the whole visible wavelength range, while the IPCE of the TPS3 film is much lower, which coincides with the observed variation trend of J_SC_ as shown in Figure 4. Moreover, the highest IPCE value for TPS2 hybrid photoanode confirms the need to control the amount of NSs in the resultant film for marked improvement in the photovoltaic performance of DSCs. Generally, IPCE is regarded as a comprehensive result related to light-harvesting efficiency, electron-injection efficiency, and charge-collection efficiency [11,36,37]. However, in view of the same charge-injection efficiency for the studied SnO_2_/dye system, it is reasonable to presume in this work that the IPCE improvement is closely related to light-harvesting and charge-collection efficiency of the photoanode film, the former of which is proportional to the dye-loading amount and light-scattering capability, while the latter is determined by the electron-transport property [38,39]. As for TPS1 and TPS2, considering their lower light-scattering capability compared with bare SnO_2_ NA (sample TP), we believe that the higher IPCE is attributed to the significantly enhanced electron-collection efficiency and increased dye-loading amount. Regarding TPS3 and TPS4, the significantly decreased IPCE could be ascribed to the more remarkable reduction of dye loading and light scattering than the increase of electron-collection efficiency, as discussed above.

### 3.7. Schematic Views of Electron Transfer and Recombination

**Scheme 1:**
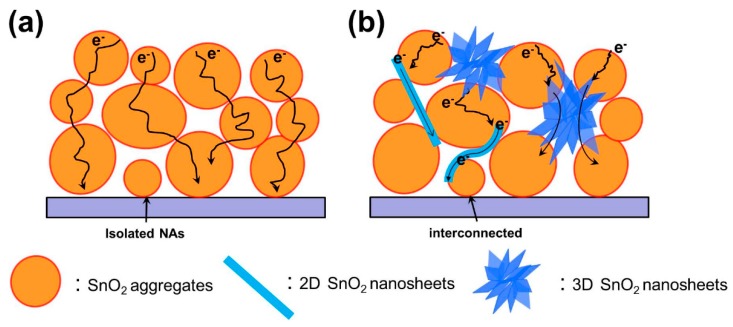
Schematic illustration of electron (e^−^) diffuse transport in (**a**) bare SnO_2_ NAs photoanode film with irregular morphology and (**b**) SnO_2_ NAs/NSs hybrid photoanode film.

When traveling across the SnO_2_ photoanode, the incident light either transmits the film or is scattered by the semiconductor oxide film in addition to the part harvested by the sensitizer. As for the typical photoanode fabricated with SnO_2_ NAs, the aggregates within the resultant film are generally submicrometer-sized and therefore are particularly effective scattering centers for visible light, giving rise to a striking increase in the light-harvesting property of the photoelectrode film. Meanwhile, since the aggregates are composed of closely packed primary nanocrystallites, the internal surface area is always reserved with the achievement of strong light-scattering capability. However, the electron transport along the 3D NAs is often retarded due to the poor interconnectivity between these microarchitectures, hence the transfer of photogenerated electrons to the conductive substrate for photocurrent extraction is not efficient (see Scheme 1a). As shown in Scheme 1b, the incorporation of NS-based architectures in the 3D NA film can not only effectively improve the interconnectivity between the aggregates but also provide additional high pathways, leading to a more favorable electron transport route and much longer diffusion length [40,41]. Therefore, the outstanding photovoltaic performance of the hybrid-based DSCs could be attributed to synergy of the received fast electron transport property from SnO_2_ NSs and the retained strong light harvesting and large surface area of SnO_2_ NAs.

## 4. Conclusions

In conclusion, a new type of SnO_2_ hybrid structure, consisting of irregular 3D SnO_2_ NAs and SnO_2_ NSs based 3D and 2D architectures, was successfully fabricated based on a function-matching strategy and subsequently applied as a multifunctional photoanode for high-efficiency DSCs. Various materials and device characterizations, including reflectance spectra, dye-absorption ability, and EIS analysis, demonstrated that the SnO_2_ hybrid films could synchronously possess the desirable features of each composition, i.e., the large dye-absorption amount and strong light-scattering property of 3D SnO_2_ NA and the excellent electron-transport characteristic of SnO_2_ NS, by controlling the ratio of NA to NS. Consequently, the DSCs fabricated with TPS2, in which 50 wt% SnO_2_ NSs was introduced in the SnO_2_ hybrid film, achieved the highest PCE of 5.59%, a remarkable 54.8% improvement compared to that of the corresponding cell fabricated with bare SnO_2_ NAs photoanode (sample TP). This work demonstrates that rationally designing a hybrid photoelectrode film could be a promising approach to enhance the efficiency of SnO_2_-based DSCs.

## Figures and Tables

**Table 1:** Characteristics of the SnO_2_ photoanodes together with the corresponding photovoltaic parameters. FF, PCE, power conversion efficiency.

Sample	J_SC_ (mA/cm²)	V_OC_ (V)	FF	PCE	Adsorbed Dye (×10^−7^ mol·cm^−2^)	R_ct_
TP	15.12	0.537	43.1	3.50	1.94	38.9
TPS1	14.61	0.640	51.6	4.85	2.23	27.8
TPS2	16.14	0.650	53.3	5.59	2.07	18.5
TPS3	11.46	0.665	58.8	4.48	1.76	11.6
TPS4	6.59	0.693	68.1	3.11	1.15	9.8

## Supplementary Materials

The following are available online at http://www.mdpi.com/1996-1944/11/9/1774/s1, Figure S1: SEM image of SnO_2_ NAs.
