# Supplementary figures and images for: Design of SnO2 Aggregate/Nanosheet Composite Structures Based on Function-Matching Strategy for Enhanced Dye-Sensitized Solar Cell Performance
Source: Materials (Basel). 2018 Sep 19;11(9):1774. doi: 10.3390/ma11091774 (PMC6164877; doi:10.3390/ma11091774)

## Supplementary Materials

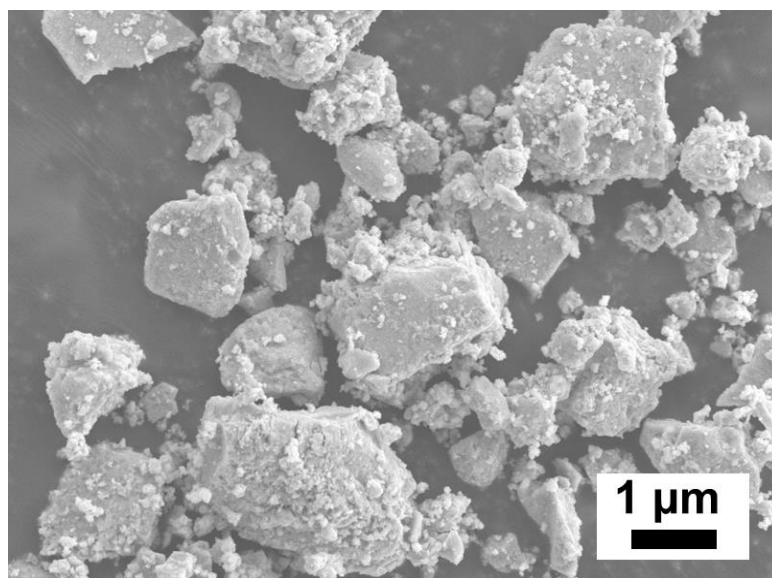

**Figure S1.** SEM images of SnO<sub>2</sub> aggregates.

Supplement: Supplementary file 1 [file materials-11-01774-s001.pdf]
